# Reinforcing effects of fentanyl analogs found in illicit drug markets

**DOI:** 10.1007/s00213-024-06641-6

**Published:** 2024-07-05

**Authors:** Alexander D. Maitland, Shelby A. McGriff, Grant C. Glatfelter, Charles W. Schindler, Michael H. Baumann

**Affiliations:** Designer Drug Research Unit (DDRU), National Institute on Drug Abuse (NIDA), Intramural Research Program (IRP), 333 Cassell Drive, Suite 4400, Baltimore, MD 21224 USA

**Keywords:** Fentanyl, Fentanyl analog, Mu-opioid receptor, Opioid, Reinforcement, Self-administration

## Abstract

**Rationale:**

The potent synthetic opioid fentanyl, and its analogs, continue to drive opioid-related overdoses. Although the pharmacology of fentanyl is well characterized, there is little information about the reinforcing effects of clandestine fentanyl analogs (FAs).

**Objectives:**

Here, we compared the effects of fentanyl and the FAs acetylfentanyl, butyrylfentanyl, and cyclopropylfentanyl on drug self-administration in male and female rats. These FAs feature chemical modifications at the carbonyl moiety of the fentanyl scaffold.

**Methods:**

Sprague-Dawley rats fitted with intravenous jugular catheters were placed in chambers containing two nose poke holes. Active nose poke responses resulted in drug delivery (0.2 mL) over 2 s on a fixed-ratio 1 schedule, followed by a 20 s timeout. Acquisition doses were 0.01 mg/kg/inj for fentanyl and cyclopropylfentanyl, and 0.03 mg/kg/inj for acetylfentanyl and butyrylfentanyl. After 10 days of acquisition, dose-effect testing was carried out, followed by 10 days of saline extinction.

**Results:**

Self-administration of fentanyl and FAs was acquired by both male and female rats, with no sex differences in acquisition rate. Fentanyl and FAs showed partial inverted-U dose-effect functions; cyclopropylfentanyl and fentanyl had similar potency, while acetylfentanyl and butyrylfentanyl were less potent. Maximal response rates were similar across drugs, with fentanyl and cyclopropylfentanyl showing maximum responding at 0.001 mg/kg/inj, acetylfentanyl at 0.01 mg/kg/inj, and butyrylfentanyl at 0.003 mg/kg/inj. No sex differences were detected for drug potency, efficacy, or rates of extinction.

**Conclusions:**

Our work provides new evidence that FAs display significant abuse liability in male and female rats, which suggests the potential for compulsive use in humans.

**Supplementary Information:**

The online version contains supplementary material available at 10.1007/s00213-024-06641-6.

## Introduction

The potent synthetic opioid fentanyl, and its various analogs, continue to drive opioid-related overdose fatalities, burdening public health resources (Scholl et al. 2018; Mattson et al. 2021). Fentanyl and structurally related fentanyl analogs (FAs) frequently appear in illicit drug markets as standalone products, heroin adulterants, or ingredients in counterfeit pain medications (Singh et al. 2019; O’Donnell et al. 2020). Like fentanyl, most FAs are selective for the mu-opioid receptor subtype (MOR) over kappa- and delta-opioid receptor subtypes, but the drugs can vary in binding affinities, potencies, efficacies, and side effects when compared to fentanyl (Armenian et al. 2018; Baumann et al. 2018; Varshneya et al. 2019, 2021; Eshleman et al. 2020). Agonist activity at MOR is responsible for therapeutic effects of fentanyl and other opioids, but this receptor also mediates adverse effects like reinforcement, physical dependence, sedation, and fatal respiratory depression (Contet et al. 2004; Comer and Cahill 2019; Varshneya et al. 2021; Varga et al. 2023). Thus, comparative studies of the pharmacological and reinforcing effects of FAs versus fentanyl are warranted.

Recent data indicate that the majority of opioid-associated deaths in the United States involve fentanyl and FAs (Tanz et al. 2024). The exact prevalence of FAs and their role in opioid-involved overdose deaths is complicated by the lack of detection with standard toxicology screens and variable cross-reactivity in fentanyl immunoassays (Stogner 2014; Schackmuth and Kerrigan 2019), thus requiring additional confirmatory analysis methods such as mass spectrometry (Prekupec et al. 2017; Cooper 2023). However, even with additional confirmatory analyses, detection of FAs can be limited by analytical challenges in distinguishing between certain structural isomers (Maher et al. 2018). Further complicating the interpretation of overdose data, most forensic samples containing FAs also contain impurities or other psychoactive drugs such as xylazine (Kariisa et al. 2021), clouding the precise role of FAs in opioid intoxications and associated deaths.

**Fig. 1:**
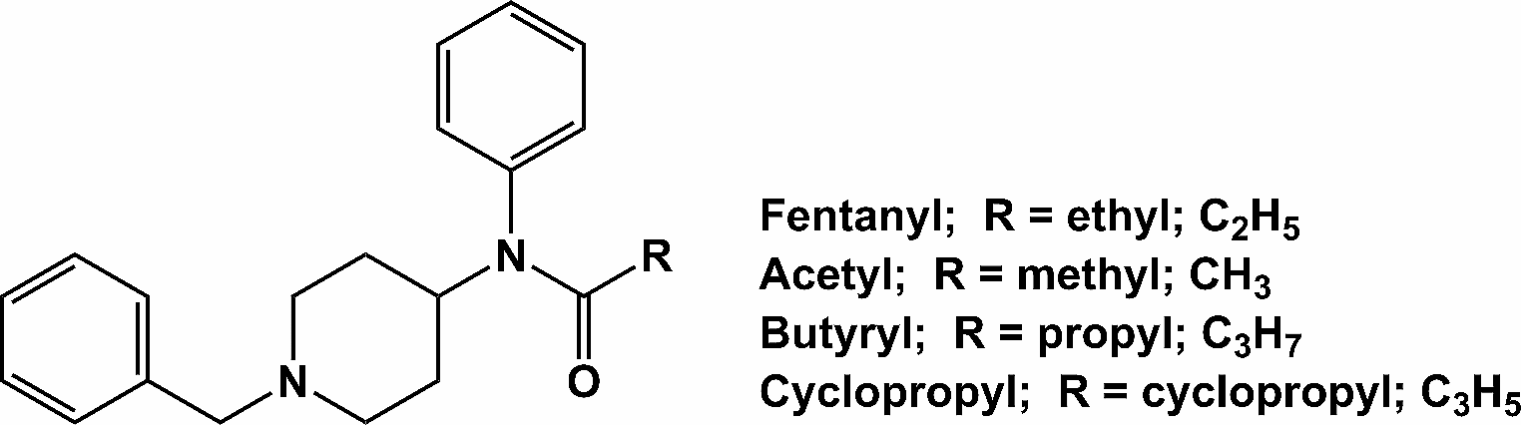
Chemical structures of fentanyl and FAs used in this study

Acetylfentanyl, butyrylfentanyl, and cyclopropylfentanyl are examples of FAs implicated in opioid overdose deaths across the United States (Lozier et al. 2015; Katselou et al. 2016; O’Donnell et al. 2017; Fogarty et al. 2018; Le and Alzghari 2019) and found in confiscated drug products (Tennyson et al. 2021). As depicted in Fig. 1, these analogs feature chemical modifications at the amide carbonyl moiety of the fentanyl scaffold, differing by the presence of a methyl (acetylfentanyl), ethyl (fentanyl), propyl (butyrylfentanyl), or cyclopropyl (cyclopropylfentanyl) group. Despite challenges to their detection, FAs appear to present a serious risk to public health (Baumann et al. 2018; O’Donnell et al. 2020) and characterizing the abuse potential of the drugs in preclinical models is critical to understanding reinforcing effects of FAs as compared to fentanyl.

Here we evaluated the effects of acetylfentanyl, butyrylfentanyl, and cyclopropylfentanyl on intravenous drug-self-administration behavior in male and female rats to characterize the structure-activity relationships for FAs relative to fentanyl. We also assessed potential sex differences in self-administration of these FAs for the first time. Previous in vitro studies show the rank-order for MOR binding affinity and potency across assay conditions to be fentanyl = cyclopropylfentanyl > butyrylfentanyl > acetylfentanyl (Astrand et al. 2020; Eshleman et al. 2020; Kanamori et al. 2022). Therefore, we hypothesized that the potency for self-administration responding of the tested FAs would follow this same rank-order. Overall, our findings show that FAs can support self-administration behavior in both male and female rats, suggesting the potential for compulsive use in humans.

## Materials and methods

### Subjects

Male and female Sprague-Dawley rats (Envigo, Frederick, MD, USA) weighing 250–350 g at the start of training were individually housed in a temperature (22.2 ± 1.1 °C) and humidity (45% ± 10%) controlled room, under a 12-hour reverse light/dark cycle (lights off 0700). Water was freely available, but food was restricted to keep weights constant or slowly increasing over the course of the study. All animal procedures were carried out in accordance with Animal Study Proposals approved by the Animal Care and Use Committee of the National Institute on Drug Abuse (NIDA), Intramural Research Program (IRP). Animal facilities were fully accredited by the American Association for Accreditation of Laboratory Animal Care.

### Surgery procedure

Male rats were anesthetized with a combination of 75 mg/kg ketamine and 5 mg/kg xylazine (i.p.). Female rats were anesthetized with either a combination of 75 mg/kg ketamine and 5 mg/kg xylazine (i.p.) or with isoflurane (1–3% at 2 L/min). Jugular catheters were surgically implanted, and the distal end of the catheter was attached to a back mount that could be connected to a fluid swivel (Instech Laboratories, Plymouth Meeting, PA, USA), as previously described (Schindler et al. 2016). Rats received ketoprofen (2.5 mg/kg, s.c., Butler Schein, Owings Mills, MD, USA) for post-operative pain relief immediately following surgery. Rats were given at least 7 days to recover from surgery before starting experimental sessions. Catheters were flushed 5 days a week with a gentamicin saline solution (0.2 mL, 5 mg/mL, Butler Schein) prepared from stock gentamicin.

### Drugs

Acetylfentanyl HCl, butyrylfentanyl HCl, and cyclopropylfentanyl HCl were generously provided by the (NIDA Drug Supply Program (Rockville, MD, USA). Fentanyl HCl was obtained from the pharmacy at the NIDA, IRP, in Baltimore, MD. Each drug was dissolved in 0.9% bacteriostatic saline to achieve the specific doses used for self-administration, and all doses are expressed as the salt form.

### Drug self-administration

Ten training chambers were used for self-administration sessions (ENV-008CT, Med Associates, St. Albans, VT, USA). Each chamber was enclosed in a sound‐attenuation cubicle equipped with a fan to provide ventilation and stable background noise. Each chamber had a grid floor and two nose‐poke response holes (ENV‐114BM, Med Associates), one on each side of a food trough (not used in this study). The nose‐poke holes were illuminated from inside the hole by a dim yellow light. Experimental events were controlled by a MED‐PC computer system (Med Associates).

Active nose poke responses resulted in delivery of a constant volume of drug (0.2 mL) over a period of 2 s on a fixed-ratio (FR) 1 schedule, followed by a 20 s timeout. Responses during the timeout were recorded but had no programmed consequences. Sessions lasted for two h every weekday. The concentration of drug in the infusion solution was adjusted according to the weight of the rat, which was taken daily. This ensured accurate mg/kg dosing upon delivery of the fixed 0.2 mL infusion volume. When weight changed by 20 g or a rat progressed onto a new dose, a new drug solution was prepared. Acquisition doses were 0.01 mg/kg/inj for fentanyl and cyclopropylfentanyl, and 0.03 mg/kg/inj for acetylfentanyl and butyrylfentanyl. These doses were chosen based on our unpublished findings examining the cardiovascular effects of the drugs in rats, and published in vitro MOR binding affinities (Baumann et al. 2018; Eshleman et al. 2020). A separate group of rats was given saline for 10 days as the self-administered substance. Rats were trained for 10 days, and a rat was considered to have acquired drug self-administration behavior by the criteria of more than 10 active responses and at least 75% active responses. Following 10 days of acquisition training, dose-effect testing began. Three additional doses were tested in a non-systematic order. Each dose was tested for only 3 days, and responses for days 2 and 3 were averaged for statistical analysis. Following dose-effect testing, the rats were returned to the training dose for 3 days and then saline was substituted for each drug for 10 days of extinction testing. We initially trained 6–13 rats per drug, however with catheter loss (assessed with a methohexital patency test) early in dose-effect training, more rats were added to some groups. As a result, not all rats were tested at all doses and group sizes varied. Final group numbers are given in figure captions.

### Statistical analysis

Data were analyzed using GraphPad Prism 10 software (GraphPad Scientific, San Diego, CA, USA). We followed the best practice recommendations for analyzing sex-based data from Rich-Edwards and Maney (2023). Acquisition data were first analyzed with the Gehan-Breslow-Wilcoxon survival analysis to compare the rate of acquisition for fentanyl and FA self-administration between sexes. Acquisition data for each sex were then analyzed using a two-way ANOVA (active vs. inactive responses x treatment day), followed by post-hoc analysis with Šídák’s multiple comparisons test. Rats were included in the acquisition analysis if they completed at least 7 days of acquisition and met acquisition criteria. Dose-response analyses were performed for each drug using dose and sex as factors for a two-way ANOVA. Post-hoc analysis was conducted using Dunnett’s multiple comparisons test, to compare the averaged active responses to the last 2 days of saline self-administration. Post-hoc analyses for sex comparisons were only considered if there was a significant dose x sex interaction. Dose–response analyses were then carried out for each sex using a two-way ANOVA, with active vs. inactive responses x treatment day as factors. Post-hoc analyses were conducted using Dunnett’s multiple comparisons test, which compared drug effects to the averaged active responses of the last 2 days of saline self-administration. For a dose to be included in the dose-effect analysis, a rat had to complete all 3 days of testing on that dose. Extinction analysis was performed using the Gehan-Breslow-Wilcoxon survival analysis to compare the rate of extinction (days until acquisition criteria were no longer met) between male and female rats, and further analyzed using a two-way ANOVA.

## Results

### Acquisition of self-administration

**Fig. 2:**
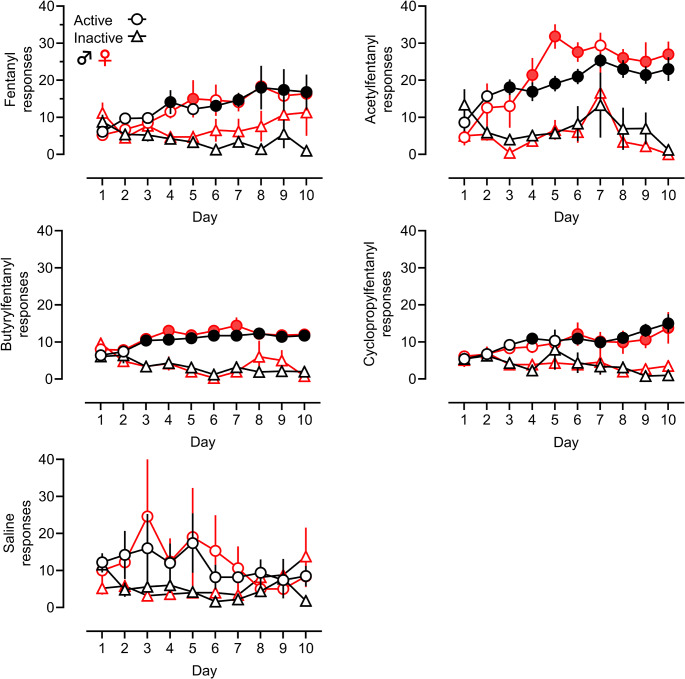
Acquisition of self-administration for fentanyl, FAs, and saline. Active (circles) and inactive (triangles) responses are shown for males (black) and females (red), with filled circles indicating significant differences from inactive responses on a given day (Šídák’s test, *p* < 0.05). Group numbers are fentanyl (male *n* = 13, female *n* = 11), acetylfentanyl (male *n* = 7, female *n* = 5), butyrylfentanyl (male *n* = 7, female *n* = 4–5), cyclopropylfentanyl (male *n* = 8–9, female *n* = 7), saline (male *n* = 5, female *n* = 5)

Figure 2 shows acquisition data for fentanyl (0.01 mg/kg/inj), butyrylfentanyl (0.03 mg/kg/inj), cyclopropylfentanyl (0.01 mg/kg/inj), and acetylfentanyl (0.1 mg/kg/inj) in male and female rats. Active and inactive responses are shown, while timeout responses are not. Fentanyl and all three FAs produced acquisition of self-administration, and the survival analysis did not reveal any sex differences in the rate of acquisition of self-administration for fentanyl (χ2 = 0.4778, d.f.=1, *p* = 0.4894), acetylfentanyl (χ2 = 1.319, d.f.=1, *p* = 0.2507), butyrylfentanyl (χ2 = 0.1097, d.f.=1, *p* = 0.7405), or cyclopropylfentanyl (χ2 = 0.2928, d.f.=1, *p* = 0.5884). Since the survival analysis demonstrated no significant effect of sex for any of the treatment groups, we combined the male and female acquisition data for each drug and plotted these data, as depicted in Supplementary Figure S1.

A two-way ANOVA for the acquisition data in male subjects revealed significant main effects of active vs. inactive responding for fentanyl (F[1, 234] = 64.03, *p* < 0.0001), butyrylfentanyl (F[1, 118] = 194.0, *p* < 0.001), cyclopropylfentanyl (F[1, 152] = 63.35, *p* < 0.0001), acetylfentanyl (F[1, 117] = 63.15, *p* < 0.001), and saline (F[1, 78] = 12.56, *p* < 0.001). The analysis also demonstrated a significant interaction of response x treatment day for fentanyl (F[9, 234] = 2.53, *p* < 0.0086), butyrylfentanyl (F[9, 118] = 5.22, *p* < 0.0001), and cyclopropylfentanyl (F[9, 152] = 3.33, *p <* 0.0009), but not for acetylfentanyl (F[9, 117] = 1.95, *p =* 0.0515) or saline (F[9, 78] = 0.544, *p* = 0.8377). Šídák’s post-hoc test showed that active responses in male rats were significantly greater than inactive responses on the last few days of training for all drug treatments but not for saline (see filled black circles in Fig. 2).

A two-way ANOVA for the acquisition data in female subjects revealed significant main effects of active vs. inactive responding for fentanyl (F[1, 198] = 11.04, *p <* 0.0011), acetylfentanyl (F[1, 80] = 88.88, *p* < 0.0001), butyrylfentanyl (F[1, 74] = 117.9, *p* < 0.0001), cyclopropylfentanyl (F[1, 118] = 35.28, *p* < 0.0001), and saline (F[1, 78] = 4.903, *p <* 0.0297). The analysis also demonstrated a significant interaction of response x treatment day for acetylfentanyl (F[9, 80] = 2.379, *p <* 0.0193), butyrylfentanyl (F[9, 74] = 4.037, *p <* 0.0003), cyclopropylfentanyl (F[9, 74] = 4.037, *p <* 0.0003), but not for fentanyl (F[9, 198] = 1.087, *p* = 0.3740) or saline groups (F[9, 78] = 0.9462, *p* = 0.4907). Šídák’s post-hoc test showed that active responses for female rats were significantly greater than inactive responses on the last few days of training for all drug treatments, with the exception of fentanyl (see filled red circles in Fig. 2). In the case of fentanyl acquisition in female rats, an elevated level of inactive nose poke responding by certain subjects on days 8, 9, and 10 eliminated significant differences between active vs. inactive responding on those days.

### Dose-effect testing

**Fig. 3:**
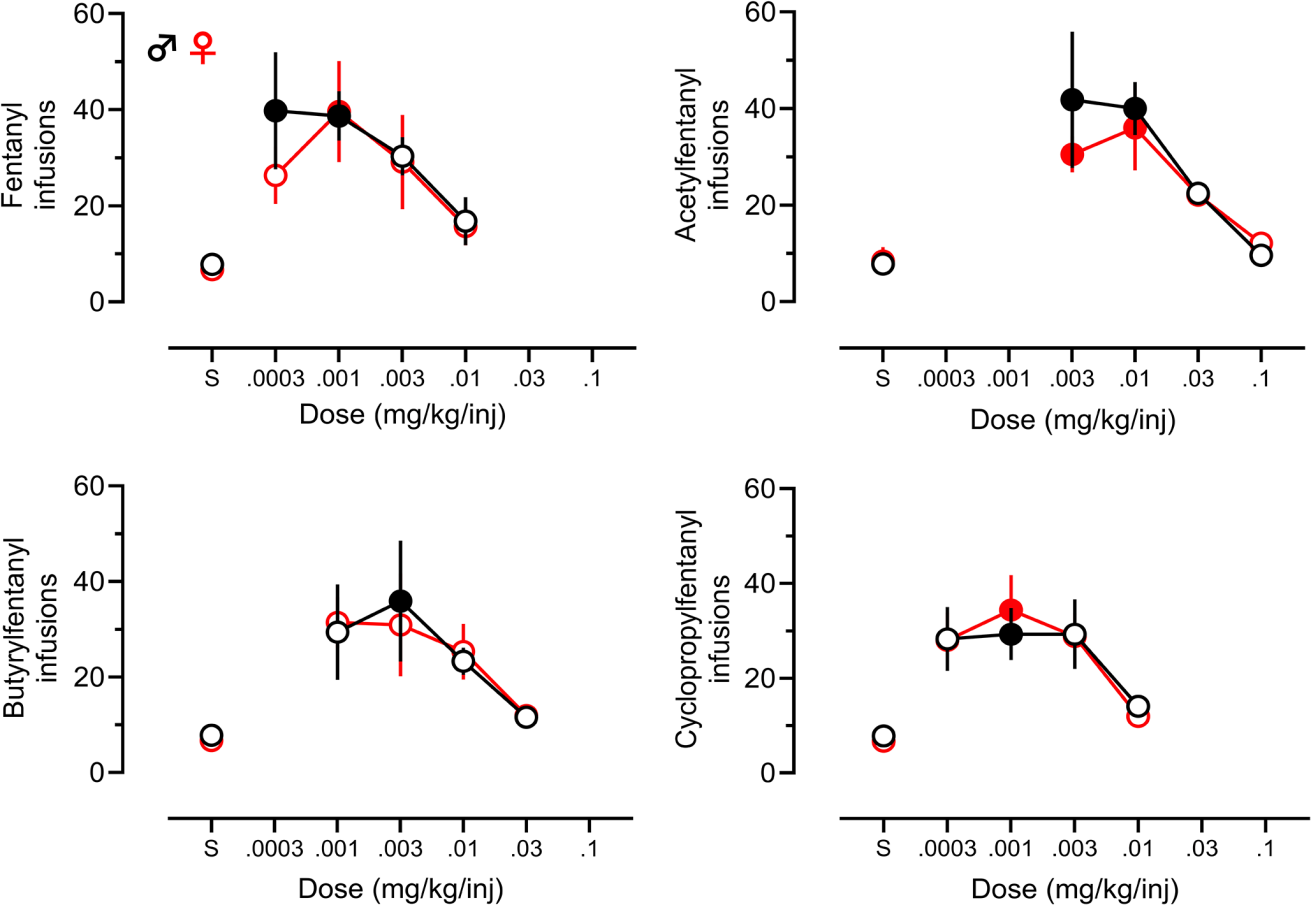
Dose-effect curves for fentanyl and FAs compared to the last two days of saline. Filled symbols indicate significant differences from saline (Šídák’s test, *p* < 0.05). Active responses shown for males (black) and females (red), with filled circles indicating a significant difference from saline responses. Group numbers are fentanyl (male *n* = 5–13, female *n* = 8), acetylfentanyl (male *n* = 5–7, female *n* = 4–5), butyrylfentanyl (male *n* = 5–7, female *n* = 4), cyclopropylfentanyl (male *n* = 5–9, female *n* = 5–6)

Following acquisition, animals continued to dose-effect testing. In Fig. 3, dose-response data for the average number of active responses in the last 2 days of testing are plotted, along with the average responses for the last 2 days of saline training. Fentanyl and all three tested FAs produced partial inverted U-shaped dose-effect functions, most likely due to the absence of doses on the ascending limb of the dose-response curve for each drug. For all drugs tested, a significant main effect of dose was observed (fentanyl F[4, 75] = 6.864, *p* < 0.0001; acetylfentanyl F[4, 44] = 12.33, *p* < 0.0001; butyrylfentanyl F[4, 40] = 5.816, *p <* 0.0009; and cyclopropylfentanyl F[4, 50] = 9.454, *p* < 0.0001), but there was no significant main effect of sex, nor a dose x sex interaction. Since we found no effect of sex on the dose-response functions, we combined the male and female data and re-plotted the dose-effect results, as depicted in Supplementary Figure S2. When the data are combined in this manner, the evidence for inverted U-shaped dose-response curves is better illustrated for fentanyl, butyrylfentanyl, and cyclopropylfentanyl.

Post-hoc analyses for the dose-response data revealed some subtle sex-specific effects, with significant differences from saline within each sex noted by filled symbols in Fig. 3. For fentanyl responding, males showed a significant difference from saline at 0.0003 mg/kg/inf (*p* = 0.0291) and 0.001 mg/kg/inf (*p* = 0.0092), while females showed a significant difference from saline only at 0.001 mg/kg/inf (*p* = 0.0113). For acetylfentanyl, males showed significant differences from saline at 0.003 mg/kg/inf (*p* = 0.0006) and 0.01 mg/kg/inf (*p* = 0.0004), and females similarly showed significant differences at 0.003 mg/kg/inf (*p* = 0.0320) and 0.01 mg/kg/inf (*p* = 0.0034). For butyrylfentanyl, males showed a significant difference from saline at 0.003 mg/kg/inf (*p* = 0.0156), whereas females did not show a significant difference from saline at any dose, perhaps due to higher variability in response rates for this drug. For cyclopropylfentanyl, males showed significant differences from saline at 0.0003 mg/kg/inf (*p* = 0.0252), 0.001 mg/kg/inf (*p* = 0.0139), and 0.003 mg/kg/inf (*p* = 0.0248), and females showed significant differences at 0.0003 mg/kg/inf (*p* = 0.0268), 0.001 mg/kg/inf (*p* = 0.0016), and 0.003 mg/kg/inf (*p* = 0.0145).

### Extinction testing

**Fig. 4:**
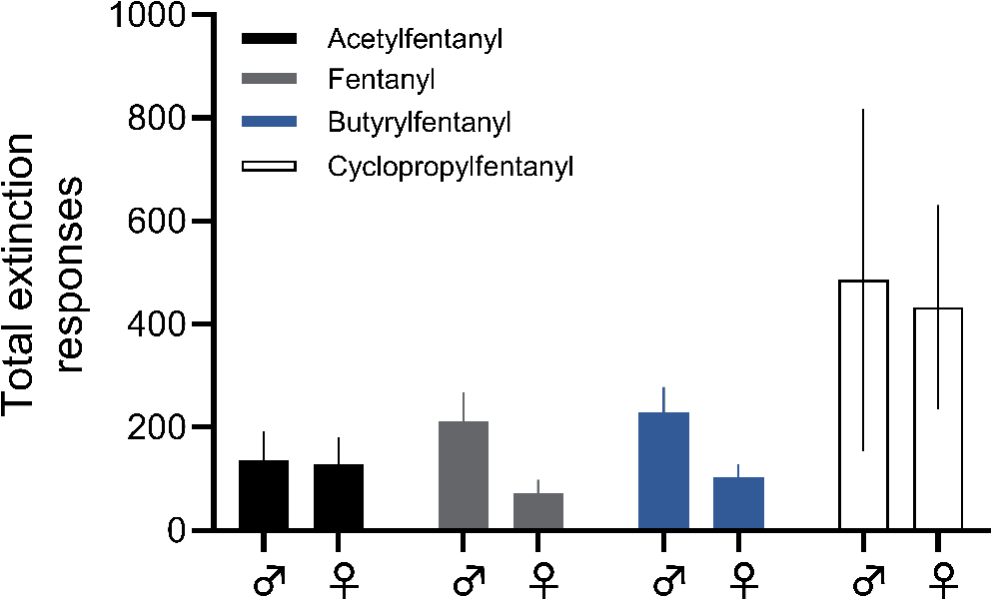
Total responses across 10 days of extinction testing for fentanyl (male *n* = 7, female *n* = 3), acetylfentanyl (male *n* = 6, female *n* = 4), butyrylfentanyl (male *n* = 3, female *n* = 4), cyclopropylfentanyl (male *n* = 4, female *n* = 5)

Following dose-effect testing, animals continued on to extinction training, receiving saline infusions for active responses (Fig. 4). The survival analysis revealed no sex differences in the rate of extinction of self-administration for any drug (fentanyl χ2 = 1.038, d.f.=1, *p* = 0.3082; acetylfentanyl χ2 = 0.7786, d.f.=1, *p* = 0.3776; cyclopropylfentanyl χ2 = 0.1566, d.f.=1, *p* = 0.6923). Extinction testing for butyrylfentanyl featured only a single non-censored male animal; thus, survival analysis was not conducted for this drug. While cyclopropylfentanyl appeared to support higher extinction responding for both sexes, further analysis of total extinction active responses revealed no significant effects for any drugs. Specifically, two-way ANOVA demonstrated no effect of drug (F[3,27] = 2.386, *p* = 0.0911), no effect of sex (F[1, 27] = 0.5764, *p* = 0.4543) and no sex x drug interaction (F[3,27] = 0.08443, *p* = 0.9680). Since survival analysis revealed no significant effect of sex on extinction, we replotted the combined extinction data, as depicted in Supplementary Figure S3.

## Discussion

No studies to date have examined the reinforcing effects of the FAs acetylfentanyl, butyrylfentanyl, and cyclopropylfentanyl, as compared to the parent compound, fentanyl. The present study examined whether these clandestine FAs support i.v. drug self-administration behavior compared to fentanyl. Our findings show for the first time that all three tested FAs support i.v. drug self-administration behavior in both male and female rats, suggesting potential fentanyl-like abuse liability in humans. We hypothesized that self-administration responding for these FAs would follow the general rank order for in vitro MOR binding affinity, potency, and efficacy, with cyclopropylfentanyl producing equivalent self-administration responding to fentanyl. Indeed, maximum responding during dose-effect testing followed the same rank-order of potency for both sexes, with comparable potencies for cyclopropylfentanyl and fentanyl, lower potency for butyrylfentanyl, and the lowest potency for acetylfentanyl.

Both acetylfentanyl and butyrylfentanyl produced partial inverted U-shaped dose-effect functions that were right shifted from fentanyl, with cyclopropylfentanyl producing similar levels of active responding across doses to fentanyl. We observed an expected similarity in the levels of responding for fentanyl and cyclopropylfentanyl, with fewer doses significantly above saline for acetylfentanyl and butyrylfentanyl. However, it should be noted that a limitation of the present study is the lack of drug doses on the ascending limb of the dose-effect functions, which prevents the determination of potency metrics (such as ED_50_). Future studies should examine the relative elasticities of demand for these FAs by generating behavioral economic demand curves (Hammerslag et al. 2020; McConnell et al. 2021), and should further interrogate reinforcing strength using progressive ratio schedules in preclinical self-administration studies (Hodos 1961; Stafford et al. 1998; Panlilio and Schindler 2000).

Prior studies show that cyclopropylfentanyl is similar to fentanyl in its binding affinity, potency, and efficacy at MOR (Eshleman et al. 2020; Xie et al. 2023). Butyrylfentanyl displays somewhat lower MOR affinity and potency compared to fentanyl, but has significantly lower efficacy (Eshleman et al. 2020; Hassanien et al. 2020). Acetylfentanyl has a 32-fold lower affinity for MOR compared to fentanyl, and significantly lower potency and efficacy at MOR compared to fentanyl (Eshleman et al. 2020). In vivo studies in mice reveal that cyclopropylfentanyl is a potent antinociceptive and respiratory depressive agent with similar potency to fentanyl, suggesting high risk for adverse effects in human users exposed to the drug (Xie et al. 2023; Varshneya et al. 2023a). Furthermore, molecular dynamic simulations show that cyclopropylfentanyl displays a binding pose in the MOR orthosteric site similar to fentanyl, whereas increasing alkyl chain length at the carbonyl moiety prevents optimal binding interactions (e.g., such as extending to a butyl chain length in valerylfentanyl) (Xie et al. 2023), and generally reduces FA potency and efficacy (Varshneya et al. 2023a).

Preclinical findings regarding the abuse liability of many newly emerging FAs is limited, especially in female animals. Varshneya and colleagues (2023b) showed that numerous FAs, including cyclopropylfentanyl, elicit dose-dependent hyperlocomotion in male mice, similar to reference MOR agonist such as morphine and fentanyl. Notably, cyclopropylfentanyl (1 mg/kg, s.c.) produced hyperlocomotion similar to the most efficacious dose of fentanyl (1 mg/kg s.c.). It is difficult to discern how other relevant factors may impact on reinforcing effects of these drugs observed in our experiments. Few preclinical studies have compared the pharmacokinetics of fentanyl and various FAs. Ohtsuka et al. (2007) reported a fentanyl half-life of 50.4 ± 5.4 min after i.v. administration of 0.03 mg/kg to rats, whereas Bergh et al. (2021) found a cyclopropylfentanyl half-life of 115 ± 11 min after subcutaneous administration of the same 0.03 mg/kg dose. As far as we are aware, the half-lives of acetylfentanyl and butyrylfentanyl are not known. Drug lipophilicity may impact drug reinforcing effects and abuse liability, with greater lipophilicity allowing more rapid diffusion through the blood-brain barrier, therefore reducing the interval between drug administration and the onset of its effects. Cyclopropylfentanyl likely has similar lipophilicity to fentanyl, though solubility data is limited. Acetylfentanyl and butyrylfentanyl have somewhat lower and higher respective log P values when compared to fentanyl (Schackmuth and Kerrigan 2023).

The present study found no substantial sex differences in drug self-administration behavior for fentanyl and the tested FAs. Our findings contrast with reports showing that females acquire i.v. self-administration faster than males across different MOR agonists, including heroin, morphine, remifentanil, and fentanyl (Lynch and Carroll 1999; Carroll et al. 2001, 2002; Cicero et al. 2003; Thorpe et al. 2020; Malone et al. 2021). Our sex-based comparisons may be limited by unbalanced numbers of males and females in each drug group, and sex differences across drugs are more likely to emerge at lower doses, potentially due to ceiling effects (Carroll and Lynch 2016). It seems possible that our study underestimated potential sex differences in drug responding because we failed to include low drug doses on the ascending limb of the dose-effect functions for fentanyl and the FAs tested. However, it is unclear if such low opioid doses would be sufficient to produce robust active responding. Furthermore, sex comparisons may be limited without more robust multidimensional behavioral analyses (Siciliano 2019). For example, the emergence of sex differences in fentanyl self-administration are highly dependent on the schedule of reinforcement, and the presence of alternative reinforcers like food (Townsend et al. 2019).

Similar levels of extinction of self-administration behavior were observed across drugs. Cyclopropylfentanyl appeared to support higher extinction active responses for both sexes, though this was not significant due to high variability in the data for this group. Given the prevalence of FAs in overdose deaths in humans, future studies should examine these drugs in an extinction-reinstatement paradigm to better characterize relapse to drug use after a period of abstinence. The reinforcing effects demonstrated in the present study highlight the unique threat posed by synthetic FAs to public health. Our work adds to the converging preclinical in vitro and in vivo evidence suggesting that these FAs may possess significant abuse liability in humans.

## Electronic supplementary material

Below is the link to the electronic supplementary material.

Supplementary Material 1
