# Supplementary material for: Reinforcing effects of fentanyl analogs found in illicit drug markets
Source: Psychopharmacology (Berl). 2024 Jul 5;241(11):2375–83. doi: 10.1007/s00213-024-06641-6 (PMC11513704; doi:10.1007/s00213-024-06641-6)
Supplement: Supplementary file 1 — Supplementary Material 1 [file 213_2024_6641_MOESM1_ESM.docx]

**SUPPLEMENTARY INFORMATION**

**Reinforcing effects of fentanyl analogs found in illicit drug markets**

Alexander D. Maitland, Shelby A. McGriff, Grant C. Glatfelter, Charles W. Schindler, and Michael H. Baumann

*Designer Drug Research Unit, National Institute on Drug Abuse, Intramural Research Program, Baltimore, Maryland*

**
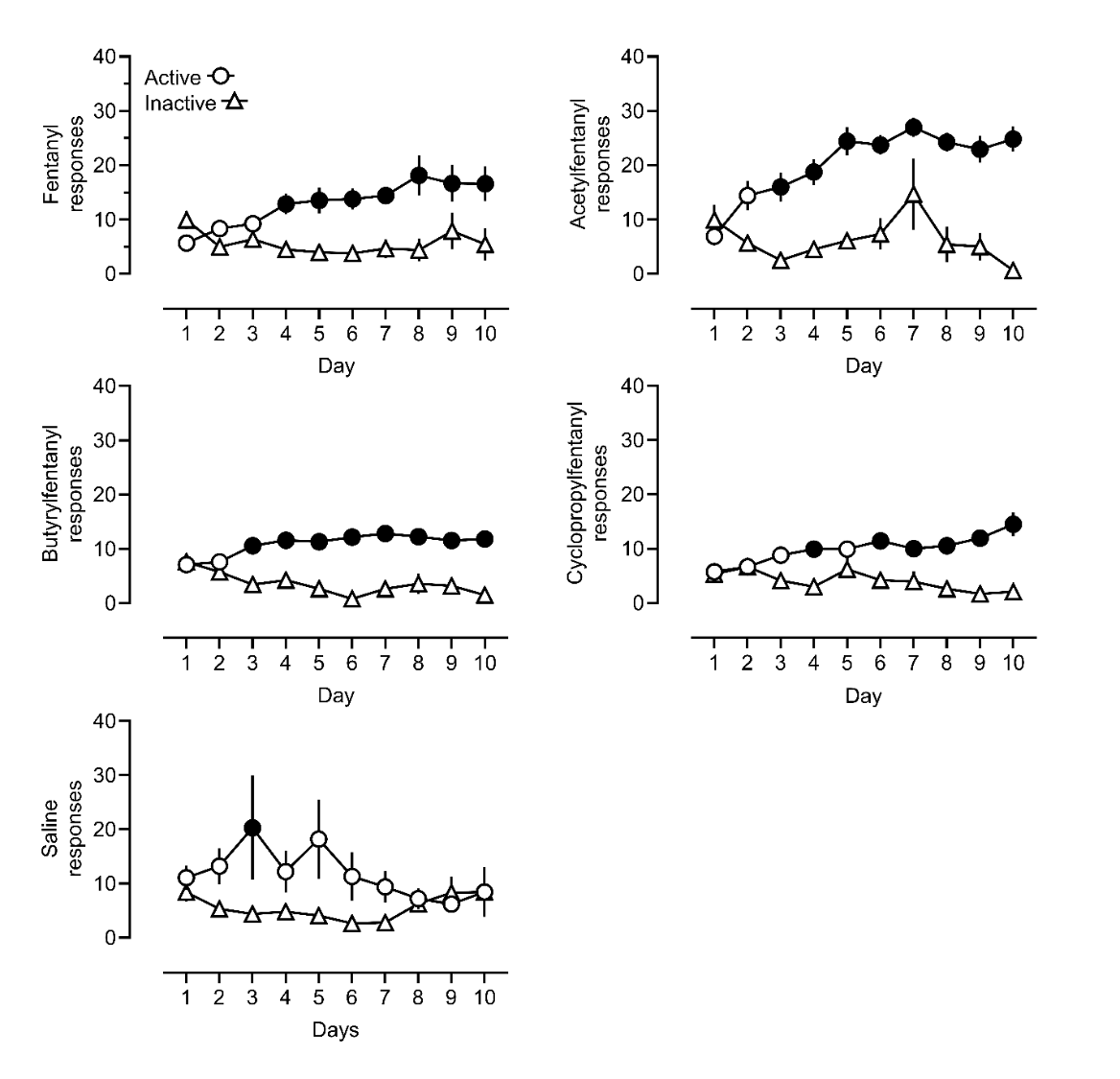
**

**Fig. S1:** Combined male and female acquisition of self-administration for fentanyl, FAs, and saline. Active (circles) and inactive (triangles) responses are shown, with filled circles indicating a significant difference from inactive responses on that day. Fentanyl (*n*=24), acetylfentanyl (male *n*=12), butyrylfentanyl (male *n*=11-12), cyclopropylfentanyl (male *n*=16-17), saline (male *n*=10).

A two-way ANOVA for the combined acquisition data from male and female subjects revealed significant main effects of active versus inactive responding for fentanyl (F[1, 450]=61.56, *p*<0.0001), acetylfentanyl (F[1, 217]=150.2, p<0.0001), butyrylfentanyl (F[1, 210]=312.6, *p*<0.0001), cyclopropylfentanyl (F[1, 290]=102.5, *p*<0.0001), and saline (F[1, 176]=14.95, *p*=0.0002). The analysis also demonstrated a significant interaction of response x day for fentanyl (F[9, 450]=3.161, *p*=0.0010), acetylfentanyl (F[9, 217]=4.030, *p*<0.0001), butyrylfentanyl (F[9, 210]=8.479, *p*<0.0001), and cyclopropylfentanyl (F[9, 290]=4.374, *p*<0.0001), but not for saline (F[9, 176]=1.368, *p*=0.2060). Šídák’s post-hoc test showed that active nose poke responses were significantly greater than inactive responses on the last few days of training for all drug treatments but not for saline (see filled black circles).


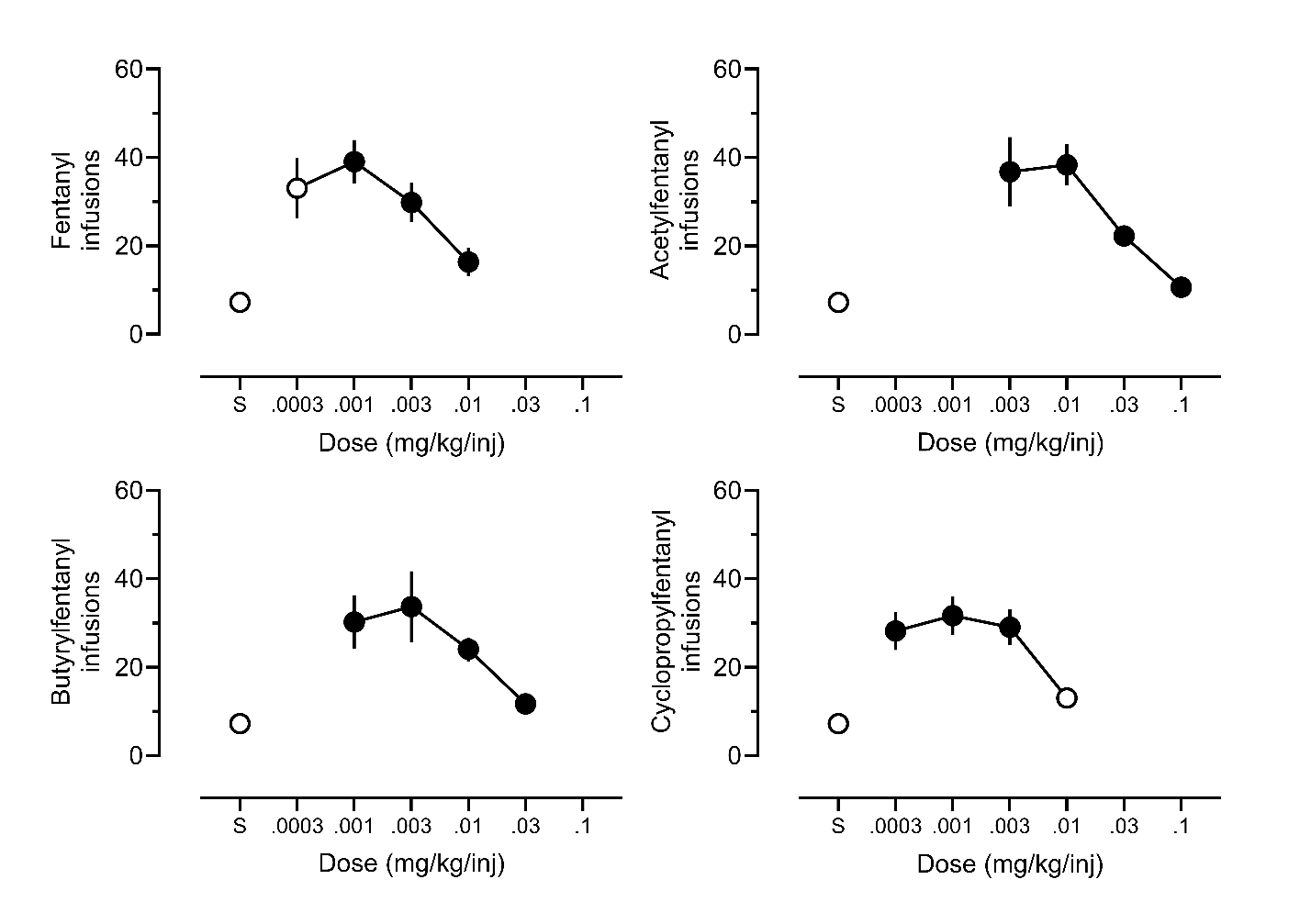


**Fig. S2:** Combined male and female dose-effect curves for fentanyl and three FAs compared to last two days of saline. Active (circles) and inactive (triangles) responses are shown, with filled circles indicating a significant difference from saline responses. Fentanyl (*n*=22-24), acetylfentanyl (*n*=11-12), butyrylfentanyl (*n*=10-12), cyclopropylfentanyl (*n*=14-16).

Dose-effect data for males and females were combined. For all drugs tested, a significant effect of dose was observed (fentanyl F[2.511, 35.78]=11.46, *p* <0.0001; acetylfentanyl F[1.592, 14.33]=16.54, *p*<0.0003; butyrylfentanyl F[1.724, 14.65]=8.837, *p*<0.0040; cyclopropylfentanyl F[2.856, 28.56]=10.69, *p* <0.0001). For fentanyl, there was a significant difference from saline at 0.001 mg/kg/inf (*p*=0.0070), 0.003 mg/kg/inf (*p*=0.0072), and 0.01 mg/kg/inf (*p*=0.0110). For acetylfentanyl, there was a significant difference from saline at 0.003 mg/kg/inf (*p*=0.0482), 0.01 mg/kg/inf (*p*=0.0029), 0.03 mg/kg/inf (*p*=0.0004), and 0.1 mg/kg/inf (*p*=0.0183). For butyrylfentanyl, there was a significant difference from saline at 0.001 mg/kg/inf (*p*=0.0306), 0.003 mg/kg/inf (*p*=0.0421), 0.01 mg/kg/inf (*p*=0.0029), and 0.03 mg/kg/inf (*p*=0.0473). For cyclopropylfentanyl, there was a significant difference from saline at 0.0003 mg/kg/inf (*p*=0.0080), 0.001 mg/kg/inf (*p*=0.0025), 0.003 mg/kg/inf (*p*=0.0045).


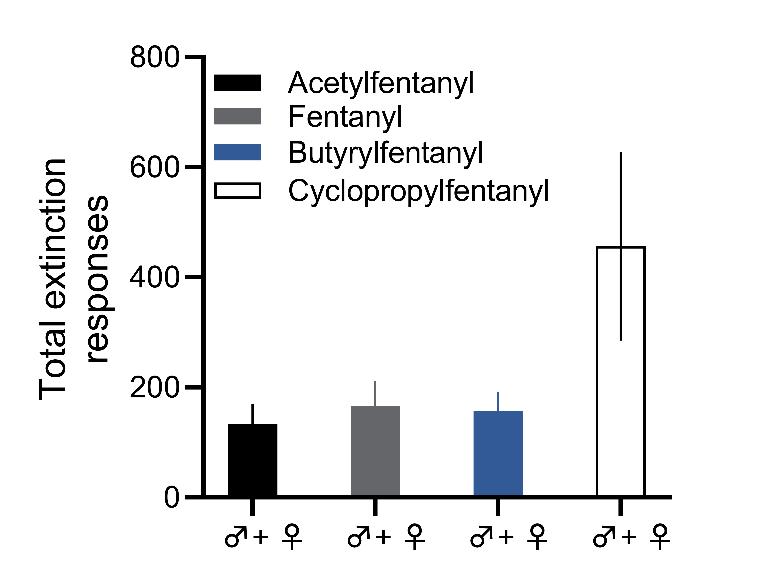


**Fig. S3:** Combined total responses for males and females across 10 days of extinction testing for fentanyl (*n*=10), acetylfentanyl (*n*=10), butyrylfentanyl (*n*= 7), cyclopropylfentanyl (*n*=9).

Total extinction active responses for males and females were combined. Total extinction active responses were not significantly different for any tested drugs (One-way ANOVA, no effect of drug F[3, 32]=2.724, *p*=0.0605).
